# Child Study

**Published:** 1896-03

**Authors:** 


### Child-Study.

There exists in the city of Chicago, and similar organizations can probably be found in other centers, a society for the promotion of child-study. While the leading idea of such study is undoubtedly psychologic, the subject is suggestive in a medical point of view, and may well be worth an editorial comment in a journal that only deals with psychologic questions in their specially medical aspects and bearings.

There is no period of life when mental and physical development is as rapid in childhood, and therefore there is none more interesting in a physiologic as well as in a psychologic point of view. Physicians have studied children in their pathologic peculiarities; pediatrics is a recognized medical specialty, but it is a reasonable question whether it might not be as well to widen its scope and take into it some attention to the unfolding of the intellectual life in its beginnings. The skilled medical practitioner can better than any one else first take note of and point out the way of correcting the morbid traits and tendencies that lead to physicial and mental degeneracy; he can study and estimate the hereditary influences and advise how they are to be met, and can instruct the mother in what should be the most fascinating pursuit of her life, the proper method of studying the development of her offspring. These are the possibilities of his profession; we do not say they are generally or even often realized.

Considering, however, only the physical side of the question of child-study, it is not a credit to our profession that while the studies of the growth and the physical data of childhood are being taken up by laymen and educators it should be in any degree behind them in the same line of investigation. While physiologists were ahead of psychologists in recognizing the value of knowlenge of the earliest developmental processes and conditions in the study of functions, it seems now that the newer school of psychologists, enlightened by the data of physiology, may in their turn put practical medicine under obligations for important facts and deductions. Sometimes they may be on the wrong track, or on one that is uncertain, but they are always suggestive and instructive in their modern methods.

The practical value of child-study should be evident to any one. The old saying that "as the twig is bent, the tree is inclined," so often quoted with a moral application, has a physical and intellectual appropriateness as well. Hence, every real acquisition of fact or legitimate theory in regard to the bodily or mental development of children has its value, and there is an ample store of such facts yet to be acquired. At the present time we may take, for example, the theories of mental and bodily degeneracy that are just now so much to the fore, and it is easy to see that they can only be proven or disproven by taking into consideration the earlier conditions of the individual and the influences that affected his development. The question as to the existence of such a type as the "born criminal" is, as might be inferred from the term itself, one that can only be settled by the study of the development and beginnings as well as the finished type; in short, by a study of the morbid tendencies and moral development of the child. As an almost purely medical line of investigation, and not the least important may be mentioned that of heredity in children, which can hardly be studied by any one so well as by the general practitioner—the family physician. Galton has laid down a plan for this line of research in his "Natural Inheritance" that is at least worthy of some consideration. The amount of valuable facts and statistics that could be obtained from a general interest in this study in the medical profession can hardly be over-estimated. Other interesting questions are some of those of the origin of insanity, especially those forms that seem to be more or less dependent upon errors of education and training and management of developmental periods, and here well-directed attention to the facts of early life will be found to be productive of valuable results. It is not meant to be understood that these questions are neglected by physicians, but more sytematic study of all the stages of early human development is needed to fully elucidate them.

It is noteworthy that this whole subject of child-study is comparatively of modern development. If we look over the bibliography as given in the report of the Commissioner of Education for 1892 and 1893, just issued from the Government press, we find comparatively few works dating back more than twenty years, and most of the papers bearing directly on the subject have appeared within the last decade. While it is likely to continue to be for the most part a specialty of educators and psychologists, it is to be hoped that our profession will not too much neglect the special opportunities it has in this particular direction.—*The Journal of the American Medical Association.*

